# The Jubilee of Anæsthesia

**Published:** 1896-10-17

**Authors:** Benjamin Ward Richardson


### Medical Progress and Hospital Clinics.

[*The Editor will be glad to receive offers of co-operation and contributions from members of the profession. All letters should be addressed to* The Editor, at the Office, 28 & 29, Southampton Street, Strand, London, W.C.]

### THE JUBILEE OF ANÆSTHESIA.

By Sir Benjamin Ward Richardson, M.D., F.R.S.

This is the jubilee year of anæsthesia. It seems impossible that fifty years should have fled away since we listened to the cries of persons operated upon, and witnessed the skill and coolness of the operator. It seems like a dream; and, indeed, it is one to those of us who have lived through the fifty years—a strange dream since the vessel known as the "Arcadia" brought the news from America to England that the moment had arrived when pain was to be set aside; when the surgeon was to do as he liked on the living subject, and no resistance was to be made to his efforts, however refined or cruel they might seem to be. In the days in which we admired the surgeon according to the dexterity of his hands and the coolness of his head, we looked upon him with a kind of awe, as if he were a dexterous, and, in some points of view, a mysterious person.

It is to be observed that the discovery of anæsthesia did not come direct from the hand of Jove, nor was it perfected in an instant as if ordered from the skies. It came, certainly, at last, with amazing rapidity, and was a phenomenon of strange appearance in the eyes of men, but it had ever been anticipated from the time when the earliest chapters of medical science were written. Dioscorides and his brother Greeks had evidently thought of it, and had made their best endeavours to accomplish it, for Dioscorides himself invented a compound which he made from the famous mandragora. This compound was afterwards commented upon by Pliny, and received the name of morion, or death wine, of which men took a dose or a cup before they were subjected to the surgeon's knife or cautery, and frequently before or during execution. The prescription was not difficult to make up if the ingredients could be obtained, and the ingredients seem to have been known. In later days mandragora was known only to the poets, who were fond of referring to the plant in their rhapsodies, and who spoke of it in terms of praise even if they did not understand its action or for what reasons the doctors had previously employed it. On my own part I obtained from the Greek islands some of the mandragora like that formerly employed; followed Dioscorides in his manner of extracting it by a weak spirit, and detected that it produced the very phenomena he had described. It led to sleep; it caused insensibility; it brought about agitation of the muscular system, leading to a kind of hysteria; and if, from the first, it had been sedulously administered with due care, it might, like the poppy, have come down to us in these days as a distinct medicine of fame and history.

Why mandragora went out of practice I never could understand, but it certainly did so, save in name. Meantime, from the days when it declined, men became masters of other substances having similar effects. They discovered they could reach the heart and blood by medicinal agents through shorter routes than are known in the process of digestion, and they began to speak of respiration as a kind of second digestion, through which the body could be influenced. They began to distil, and utilised the products of distillation, so that they made spirits of various kinds, and at last came to the conclusion, purely from experiment, that gases and vapours could be collected pneumatically, could be inhaled, and that the body could, in such manner, be brought under their influence as it is under that of the common atmosphere.

Priestley, who first discovered oxygen, was extremely alive to the matter of inhalation, so that even Mrs. Barbauld's pathetic poem, though in entire accordance with his feelings, did not prevent him from making the inquiries he thought necessary. It remained, however, for Sir Humphrey Davy to come in sight before the process of inhalation leading to anæsthesia could be considered. Davy was in every sense a far-seeing man, for he was a poet as well as a chemist, and looked at things that were to come with as much interest and pleasure as things that were. He it was who commenced the inhalation of nitrous oxide or laughing-gas; he was the first to wonder at the phenomena in the animal body that were produced by the gas; he was particularly anxious to see the utility of the gas, and was much surprised to find that it created the physiological curiosity of annulling pain as well as of exciting laughter. He thought that for the destruction of feeling or sensibility laughing-gas might be turned to account, and in the beginning of this century he actually suggested that it should be so utilised. It is a vulgar practice now to sometimes laugh at men like Davy; to say they were not practical men, and to treat their foresight with contempt, when, in fact, it is just such foresight that men follow when they begin practically to advance. The philosopher holds the lantern, and shows the way; the practical man stumbles after him, and would not even stumble in the right direction if he were not led. This was just the case in regard to anæsthesia. Humphrey Davy held up the light to his fellows, and directed their minds; Faraday some time afterwards followed Davy, and showed that the vapour of ether bore a close resemblance in its action to nitrous oxide. I have been told that Professor Turner, of University College, was singularly struck by this analogy, and that on his great day, once a year, for exhibiting the effects of laughing-gas, he would often refer to ether as the vapour nearest to the gas in its mode of producing effects. Everybody was surprised with laughing-gas, and everybody wondered why people should be made to laugh when they inhaled air charged with nitrous oxide. We wonder still at this result, a result purely physiological in character, but as yet altogether unexplainable. The administration naturally ranked as a joke and entered into research as a kind of play incident to a lecture, by which means it became popular, and one of the best songs that was possibly ever written was based upon it, and often sung with great hilarity by the late Mr. Prideaux, Q.C., to whom, as a songster, I have more than once listened. In this way laughing-gas entered on to the itinerant stage; became common in America, and was first introduced, without intention, as a kind of object lesson, into science, to be observed by audiences of minor quality until seen by one a little above the common level—Horace Wells, a dentist, of Hartford, Connecticut. Wells went to hear a lecture delivered by a Mr. Colston; went simply for the novelty of the thing, and to pass time cheerfully rather than to become a learner and teacher of any startling fact in science. He observed the three facts: (1) That nitrous oxide could be inhaled; (2) that it could make a person laugh; and (3) that it could abolish pain. He was in pain himself at the moment, and seized the last of the three facts as one that might do him personally some good. He, therefore, got another dentist friend, Dr. Riggs, to extract a tooth from him while he was insensible under the gas, and exclaimed, on recovering, "A new era in tooth-pulling." He thus set the ball rolling that was to run directly into the open field of anæsthesia. By some means Dr. Morton got to know of the details which Wells had gathered, and soon afterwards came the events which call for the present jubilee. Morton began to substitute ether for nitrous oxide, and administered vaporous ether instead of laughing-gas. It would be in vain for us to deny to Morton the credit due to him in this simple act. It has been urged that a Dr. Jackson, a friend of his, helped him in the introduction of ether, which statement may, or may not, be true. It gave origin to the introduction of a kind of patent, in the profits of which both probably intended to share, apart altogether from the ambition of discovering what might be of benefit to the whole human race. Bigelow pricked the bubble of the commercial part of the transaction, while the useful advancement went on so wonderfully that etherisation became of itself an absolute fact, accepted here through another dentist named Robinson, living in Gower Street, and may be said to have excited a sensation in science which to this day, modified as it may have been, has ever been of service to mankind.

The introduction of ether by inhalation is a wonderful fact, and is well worthy a jubilee, but I cannot at all say that in America it started in the happy way that could have been wished, and the American people seem to have taken, from the first, much the same view. The Americans are naturally proud, as a nation, at having given birth to the authors of the discovery, but they were not so gracious to the discoverers themselves as might have been anticipated. Not one of the discoverers received any award for their trouble or prescience. Wells committed suicide; Morton, after making his claims very conspicuous, practically committed the same deed; while Jackson was immured in an asylum, as though to complete the dismal character of a record which we English tested and proved with breathless hesitation. "Anæsthesia" was soon the applied word, and the phenomena relating to it exhibited in London made an actual commotion, contributed to by no less a person than the late Emperor of the French, Louis Napoleon. There is no doubt that we obtained brilliant results, and that in them we witnessed the death of pain, and some think that it would have been well if we had never changed a process to which many have gradually come back. There will be occasion for reconsideration of this curious question when the proper time for its discussion arrives.

